# Dynamics of Humic Acid, Silicon, and Biochar under Heavy Metal, Drought, and Salinity with Special Reference to Phytohormones, Antioxidants, and Melatonin Synthesis in Rice

**DOI:** 10.3390/ijms242417369

**Published:** 2023-12-11

**Authors:** Arjun Adhikari, Appiah Gregory Aneefi, Hairkham Sisuvanh, Santivong Singkham, Masele Valentine Pius, Farida Akter, Eun-Hae Kwon, Sang-Mo Kang, Youn-Ji Woo, Byung-Wook Yun, In-Jung Lee

**Affiliations:** Department of Applied Biosciences, Kyungpook National University, Daegu 41566, Republic of Korea; arjun@knu.ac.kr (A.A.); gregory22@knu.ac.kr (A.G.A.); hairkham@gmail.com (H.S.); singkham2020@gmail.com (S.S.); valentinemasele2017@gmail.com (M.V.P.); akterfarida1985@knu.ac.kr (F.A.); eunhae.kwon1@gmail.com (E.-H.K.); kmoya@hanmail.net (S.-M.K.); yjwoo2363@gmail.com (Y.-J.W.); bwyun@knu.ac.kr (B.-W.Y.)

**Keywords:** abiotic stress, environment, biostimulants, detoxification, melatonin, sustainable agriculture, toxicity

## Abstract

This study aimed to develop a biostimulant formulation using humic acid (HA), silicon, and biochar alone or in combination to alleviate the lethality induced by combined heavy metals (HM-C; As, Cd, and Pb), drought stress (DS; 30–40% soil moisture), and salt stress (SS; 150 mM NaCl) in rice. The results showed that HA, Si, and biochar application alone or in combination improved plant growth under normal, DS, and SS conditions significantly. However, HA increased the lethality of rice by increasing the As, Cd, and Pb uptake significantly, thereby elevating lipid peroxidation. Co-application reduced abscisic acid, elevated salicylic acid, and optimized the Ca^2+^ and Si uptake. This subsequently elevated the K^+^/Na^+^ influx and efflux by regulating the metal ion regulators (Si: Lsi1 and Lsi2; K^+^/Na^+^: OsNHX1) and increased the expressions of the stress-response genes OsMTP1 and OsNramp in the rice shoots. Melatonin synthesis was significantly elevated by HM-C (130%), which was reduced by 50% with the HA + Si + biochar treatment. However, in the SS- and DS-induced crops, the melatonin content showed only minor differences. These findings suggest that the biostimulant formulation could be used to mitigate SS and DS, and precautions should be taken when using HA for heavy metal detoxification.

## 1. Introduction

Climate change, rapid human population increase, and increased anthropogenic activities are the three primary causes of abiotic stresses, such as salinity, drought stress, and heavy metal toxicity, in crops [1]. The excessive application of chemical fertilizers and pesticides have increased environmental pollution, land degradation, biodiversity loss and affected human health [2]. Therefore, modern agriculture needs to use biologically safe tools and methodologies to address this issue.

Abiotic stresses in crops can jeopardize crop productivity, food security, and sustainable agriculture [3]. Soil salinization is one of the principal abiotic stresses that negatively affects global rice cultivation, affects around 20% of the arable land, and causes a decline of up to 50% in annual productivity [4]. The common cations associated with salinity are Na^+^, Ca^2+^, and Mg^2+^, while the common anions are Cl^−^, SO_4_^2−^, and HCO_3_^−^ [5]. Heavy metals are a group of elements with a high density, i.e., >5 g/cm^3^, and are considered nonessential and toxic even at low concentrations [6]. Heavy metals such as arsenic, cadmium, and lead are highly toxic to all living organisms, including humans, and result in several diseases such as cancer, kidney failure, infertility, cardiovascular diseases, and neurological disorders [1,2,7,8]. These elements induce toxicity in plants, persistency in soil, and biomagnification, which adversely affects the entire agroecosystem and food chain [9]. The global population is rapidly growing, and it is anticipated to reach nine billion by 2050. Furthermore, agricultural water requirements are expected to increase 1.3-fold by 2025 [10,11]. The occurrence of droughts is expected to reach unprecedented levels in the first half of the 21st century, which can cause a substantial decline in crop yields. In this scenario, mitigating salt stress (SS), heavy metal toxicity, and drought stress (DS) is critical for sustaining food security.

Plants adapt to various phenomena when external stress is suspected, among which chemical signaling plays a crucial role. These phenomena include ion regulation, amino acid programming, antioxidant activation, radical scavenging, and phytohormone synthesis, including melatonin, an indoleamine, which is considered a candidate phytohormone that affects the responses to various biotic and abiotic stresses [12,13,14]. The mode of action of melatonin includes genetic modification and root engineering, and because it is an amphipathic molecule, it easily diffuses into the cellular compartments and disrupts free radical chain reactions. These cause lipid peroxidation and oxidation of nucleic acids and enhance the cellular antioxidant defense mechanisms for scavenging the toxic reactive oxygen species (ROS) [4,5,15,16]. These are endogenously synthesized and consumed by the crops. Indoleamine contributes substantially to the regulation of many physiological events in animals, such as circadian rhythms, sleep, mood, body temperature, appetite, sexual behavior, retinal physiology, and the immunological system [17,18]. The mode of action of melatonin is analogous to that of auxin (indole-3-acetic acid) and has the same biosynthetic precursor, tryptophan [19,20]. The biosynthetic pathway includes the first carboxylation of l-tryptophan into tryptamine, followed by catalyzation into serotonin, which then undergoes acetylation and methylation to subsequently form *N*-acetyl-serotonin and melatonin, respectively [14,21]. Melatonin has been reported to regulate endogenous phytohormones, such as abscisic acid (ABA) and salicylic acid (SA) modulation, which influences chemical signaling in plants under stress [22]. In general, water scarcity is directly related to the rate of plant transpiration, which is triggered by ABA and proline [23]. Salinity induces a high influx of Na^+^/K^+^, resulting in an adverse effect on cell osmosis, and heavy metals intensify lipid peroxidation, resulting in cell damage. Thus, these stresses induce oxidative stress through ROS, which ultimately leads to plant death.

Humic acid (HA), silicon, and biochar are considered important tools for improving the physicochemical properties of the soil and alleviating several abiotic stresses in crops [24,25]. Biochar serves as a source of activated carbon and organic-rich material and provides interspace to sustain microorganisms, nutrients, and water. Its use has been widely reported in the removal of different organic compounds, such as dyes and pharmaceuticals, and inorganic contaminants like heavy metals [26,27]. HAs are a pool of organic carbon derived from the microorganisms and enzymatic activity of coal, peat, soil, and plant or animal remains that contain phenolic, carboxylic, and hydroxylic compounds. They play an important role in retaining water and regulating the pH and nutrient dynamics in the soil [28]. The intensification of agriculture has largely been driven by inputs derived from nonrenewable sources, such as synthetic fertilizers [29]. However, this approach has resulted in industrial pollution, deforestation, soil erosion, a decline in surface and groundwater quality, and a loss of biodiversity [30,31]. A complete substitution of inorganic fertilizers is a huge challenge. Therefore, we selected two biological tools, HA and biochar, with Si as an inorganic substitute because of its importance in alleviating several abiotic and biotic stresses in silicon hyperaccumulating crops, such as rice [6,32].

Rice is a major staple crop for more than half of the global population and sustains food security, economy, and biodiversity. Rice represents several cultures around the world and provides hope for the increasing population [33,34]. Rice requires a large amount of water to produce a quality yield. Meanwhile, water shortages and increased concentrations of heavy metals and salts can induce oxidative damage and genotoxicity in rice [35]. A sustainable approach at a higher scale is required to battle climate change and the globalization-imposed abiotic stress and toxicity in rice. In this study, we employed HA, Si, and biochar alone or in combination to evaluate their effect on rice plants that were subjected to SS, DS, and heavy metal (As, Cd, and Pb) stress conditions. Several underlying molecular aspects were analyzed with respect to the antioxidants, phytohormones modulation, gene expression, nutrient assimilation, and melatonin synthesis. From these results, we unraveled the mechanistic insights for the formulation of biological and inorganic tools to alleviate phytotoxicity in crops and for specific stress.

## 2. Results

The results are mostly described based on the findings from Experiment 5.

### 2.1. Morphological Characteristics

Experiment 1 (SS; 150 mM NaCl): Si/HA (alone or combined) had a beneficial effect on rice plant growth under normal and SS conditions.

Experiment 2 (arsenic-only stress): The presence of HA had a toxic effect on rice plant growth under As exposure. The application of biochar/HA alone or in combination had a beneficial effect under normal conditions (Appendix A).

Experiment 3 (DS): Si/HA had a beneficial effect on rice plant growth under DS conditions.

Experiment 4 (combined heavy metals [HM-C] stress: As, Cd, and Pb): Si/HA had a hazardous effect on the rice crops, especially due to the presence of HA. The application of Si alone had a positive effect on the rice plant’s growth.

Experiment 5 (SS, DS, and HM-C): Si/biochar/HA had a beneficial effect on the rice plant growth under SS and DS, whereas it had a negative effect under HM-C (As + Pb + Cd) stress.

**Figure 1:**
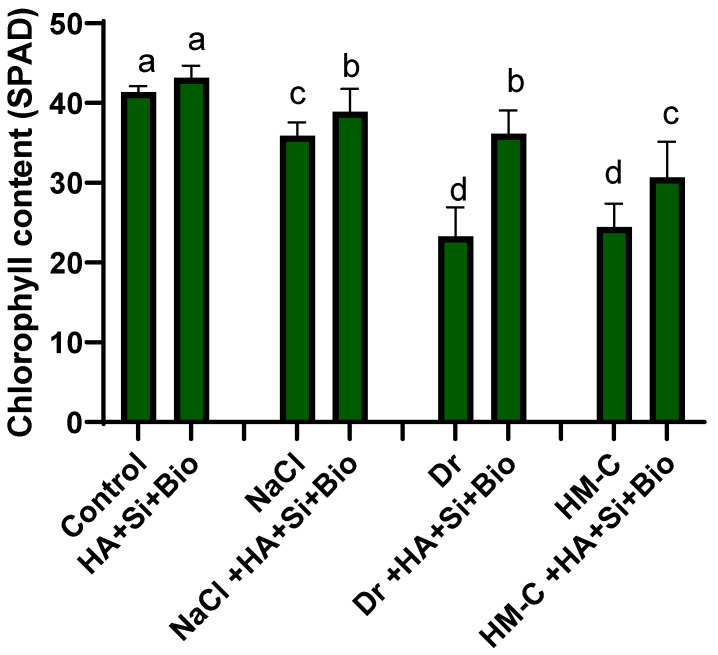
Effect of silicon, humic acid, and biochar in the chlorophyll content of rice plants under SS, DS, and HM-C. Each data point represents the mean of at least eight replicates. The bars with different letters are significantly different from each other at *p* ≤ 0.05. The abbreviations of the treatments are detailed in Table 2.

The morphological attributes, such as the tiller number, shoot weight, root weight, and shoot length, were significantly improved through the combined HA + Si + biochar treatment. In particular, when compared to the control, the SS, DS, and HM-C conditions reduced the shoot weight by 39%, 24%, and 49%, respectively. However, the HA + Si + biochar treatment increased the shoot weight by 29%, 19%, and 17% under the SS, DS, and HM-C conditions, respectively, compared to the nontreated control crops. Similar trends were observed for the root weight, shoot length, and tiller numbers. The chlorophyll content was significantly reduced in the SS, DS, and HM-C conditions by 13%, 43%, and 40%, respectively, compared to that of the control (Table 1). Nevertheless, the HA + Si + biochar treatment considerably improved the chlorophyll content by 8.4%, 55%, and 25% in the SS, DS, and HM-C conditions, respectively (Figure 1).

**Table 1:** Effect of the combined application of silicon, humic acid (HA), and biochar on the morphological attributes of rice plants grown under salt stress (NaCl), drought stress, and combined heavy metal (HM-C; arsenic, cadmium, and lead) stress conditions.

Treatment	TLN	SW (g·pot^−1^ F.W.)	RW (g·pot^−1^ F.W.)	SL (cm)
**No-Stress Condition (NS)**				
Control	15 ± 0.86 ab	46.02 ± 0.26 a	32.94 ± 0.54 b	61.2 ± 0.43 a
HA + Si + biochar	16.33 ± 0.57 a	48.63 ± 0.96 a	34.81 ± 1.07 a	61.8 ± 0.44 a
**Salt-Stressed Condition (SS)**				
NaCl	11 ± 1.32 c	27.92 ± 0.34 d	18.12 ± 0.49 e	59.46 ± 0.20 b
NaCl (HA + Si + biochar)	14 ± 0.5 b	36.15 ± 0.34 c	27.99 ± 0.39 c	61.36 ± 0.8 a
**Drought-Stressed Condition (DS)**				
Drought	14.5 ± 0.5 b	34.92 ± 2.30 c	25.35 ± 0.37 d	59.2 ± 0.45 b
Drought (HA + Si + biochar)	15.66 ± 0.76 ab	41.61 ± 1.98 b	31.92 ± 1.43 b	61.7 ± 0.43 a
**Combined Heavy Metal Stress Condition (HM-C)**				
HM-C	10.66 ± 1.6 c	23.16 ± 0.34 e	19.2 ± 1.25 e	56.16 ± 0.32 c
HM-C + HA + Si + biochar	11.66 ± 1.44 c	27.28 ± 2.82 d	24.16 ± 1.92 d	56.13 ± 0.37 c

NS: normal stress conditions, SS: NaCl stress, DS: drought stress, HM-C: combined heavy metal stress, TLN: tiller number, SW: shoot weight, RW: root weight, SL: shoot length. Each data point represents the mean of at least six replicates, and each pot consists of two plants. The columns with different letters are significantly different from each other at *p* ≤ 0.05. The abbreviations for the treatments are detailed in Table 2.

### 2.2. Physiochemical Analysis

#### 2.2.1. Quantification of Si and As and the Expression of Their Transporter Genes OsLsi1 and OsLsi2

**Figure 2:**
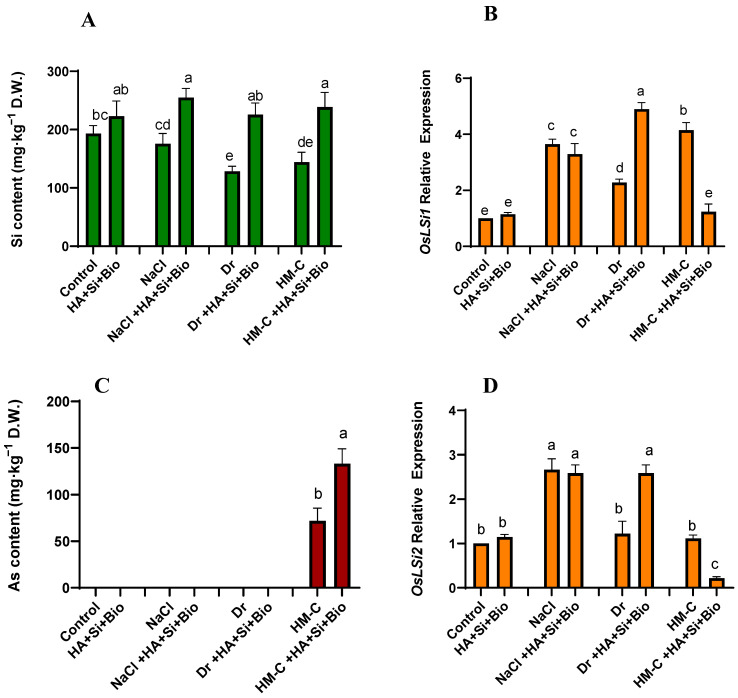
Quantification of the silicon (**A**) and arsenic content (**B**) in the rice shoots and the expression pattern of its transporter genes OsLsi1 and OsLsi2 in the rice shoots (**C** and **D**, respectively). The error bars represent the standard deviation. Each data point represents the mean of at least three replicates. The bars with different letters are significantly different from each other at *p* ≤ 0.05. ND: Not detected. The abbreviations of the treatments are detailed in Table 2.

The application of the combined treatment (HA + Si + biochar) significantly increased Si under all conditions and resulted in an increase of 15%, 50%, 75%, and 65% under the NS, SS, DS, and HM-CS conditions, respectively. The combined treatment also significantly increased the As content by 85% compared with the nontreated control crops. The Si and As transporter genes, OsLsi1 and OsLsi2, showed differential patterns of expression. Here, the Lsi1 expression was increased by 15% and 115% under the NS and DS conditions, respectively. However, a decrease of 9% and 70% was observed under the SS and HM-C conditions, respectively, when treated with HA + Si + biochar. In the case of Lsi2, the HA + Si + biochar treatment significantly decreased the expression by 80% under the HM-CS condition, whereas no difference was noted under the SS condition. In contrast, Lsi2 was significantly elevated by 111% under the DS conditions, and an increase of 15% was observed under the NS condition (Figure 2).

#### 2.2.2. Quantification of Cd and Pb and the Expression of Their Related Genes OsMTP1 and OsNramp

**Figure 3:**
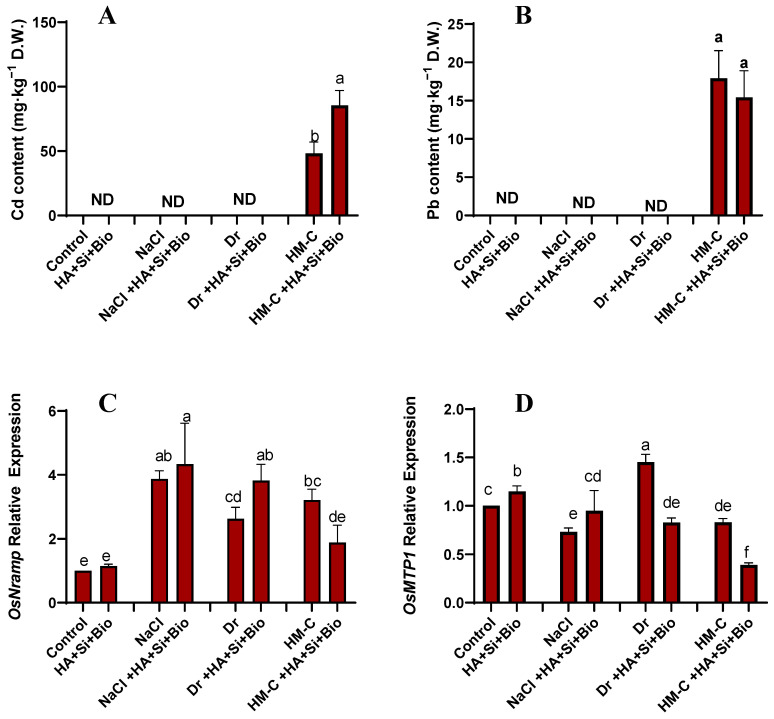
Quantification of the cadmium and lead content (**A** and **B**, respectively) and the differential expression pattern of the metal tolerance protein (OsMTP1) and natural resistance-associated macrophage protein (OsNramp) genes in the rice shoots (**C** and **D**, respectively). The error bars represent the standard deviation. Each data point represents the mean of at least three replicates. ND: Not detected. The bars with different letters are significantly different from each other at *p* ≤ 0.05.

The combined treatment (HA + Si + biochar) significantly increased the Cd content by 76%, whereas no significant differences were observed for Pb. The HA + Si + biochar treatment increased the OsNramp expression by 15%, 12%, and 45% under the NS, SS, and DS conditions, respectively, but it was significantly downregulated by 41% under the HM-C condition. The HA + Si + biochar treatment significantly downregulated the MTP1 expression by 42% and 52% under the DS and HM-CS conditions, respectively. However, considerable elevated levels were observed under the NS and SS conditions (Figure 3).

#### 2.2.3. Analysis of Na^+^/K^+^/Ca^2+^ and Its Regulator OsNHX1 Expression

HA + Si + biochar increased the Na content under the NS and DS conditions by 14% and 15%, respectively. It was significantly reduced under the SS and HM-C conditions by 41% and 27%, respectively. Similarly, the HA + Si + biochar treatment increased the K content under all conditions, except the HM-C condition, by 15%, 49%, and 48%, under the NS, SS, and DS conditions, respectively, and it was reduced by 37% under the HM-C condition.

**Figure 4:**
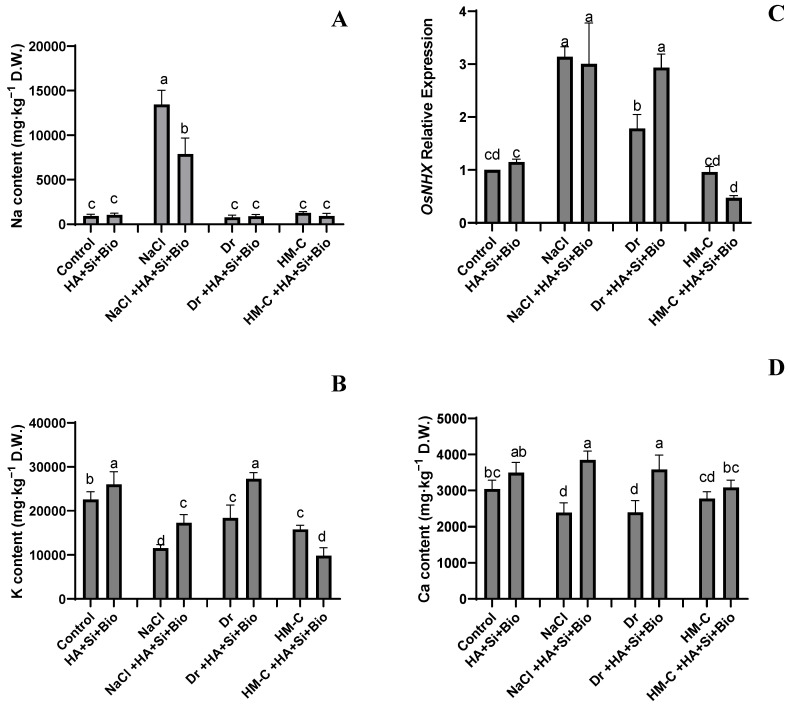
Quantification of the Na^+^/K^+^/Ca^2+^ ion concentrations (**A**, **C**, and **D**, respectively) in the rice shoots and the expression level of its regulator gene, OsNHX1 (**B**). The error bars represent the standard deviation. Each data point represents the mean of at least three replicates. The bars with different letters are significantly different from each other at *p* ≤ 0.05. The abbreviations of the treatments are detailed in Table 2.

HA + Si + biochar increased the Ca content by 14%, 61%, 49%, and 11% under the NS, SS, DS, and HM-C conditions, respectively. The HA + Si + biochar treatment increased the NHX1 expression under the NS and DS conditions by 15% and 64%, respectively. However, the expression level was reduced by 4% under the SS and 50% under the HM-C conditions (Figure 4).

#### 2.2.4. Analysis of Endogenous Phytohormones (ABA and SA)

**Figure 5:**
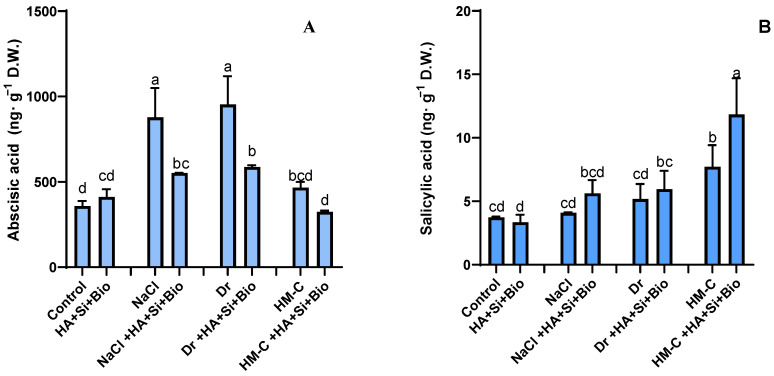
Effect of the co-application (humic acid [HA], silicon, and biochar) on the endogenous phytohormones abscisic acid (**A**) and salicylic acid (**B**) level in the rice shoots subjected to stress. Each data point represents the mean of at least three replicates. The bars with different letters are significantly different from each other at *p* ≤ 0.05. The abbreviations of the treatments are explained in Table 2.

The content of the endogenous phytohormone ABA was significantly reduced by the HA + Si + biochar treatment under the SS (37%), DS (38%), and HM-C (30%) conditions. However, an opposite trend was observed for the concentration of SA, where the HA + Si + biochar-treated plants showed a considerable elevation of 37%, 15%, and 53% under the SS, DS, and HM-C conditions, respectively. Under the NS, HA + Si + biochar treatment increased the ABA content by 15%, whereas it lowered the SA content by 10% (Figure 5).

#### 2.2.5. Quantification of the Melatonin Proline and Malondialdehyde (MDA) Levels

**Figure 6:**
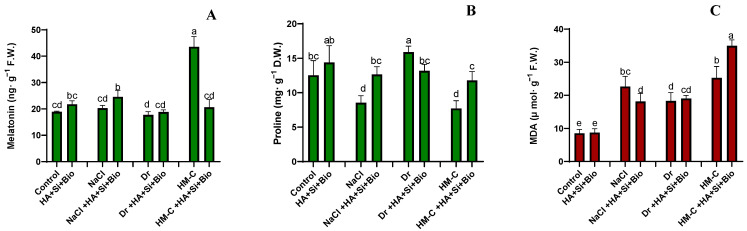
Effect of co-application on the endogenous melatonin synthesis (**A**), proline (**B**), and extent of lipid peroxidation—malondialdehyde concentration (**C**) on the rice shoot. Each data point represents the mean of at least three replicates. The bars with different letters are significantly different from each other at *p* ≤ 0.05. The abbreviations of the treatments are detailed in Table 2.

Melatonin was significantly increased by 130% under HM-C stress. However, only minor differences were observed under the SS and DS conditions compared with the control. Within the stress, the HA + Si + biochar increased the melatonin by 15% under the NS, 21% under the SS, and 6% under the DS condition. However, under the HM-C condition, the HA + Si + biochar significantly reduced the melatonin content by 50% (Figure 6A).

The highest proline content was observed in the DS-controlled plants compared to the other stresses and normal condition. Upon the HA + Si + biochar treatment, the proline content was reduced by 17% under the DS condition. However, it was increased by 14%, 47%, and 53% under the NS, SS, and HM-C conditions, respectively (Figure 6B).

The MDA content was significantly increased by 38% with the HA + Si + biochar treatment in the HM-C-stressed plants. However, under the SS condition, HA + Si + biochar decreased the MDA content by 20%. Only minor differences were observed under the NS and DS conditions (Figure 6C).

#### 2.2.6. Antioxidant Activity Assay

**Figure 7:**
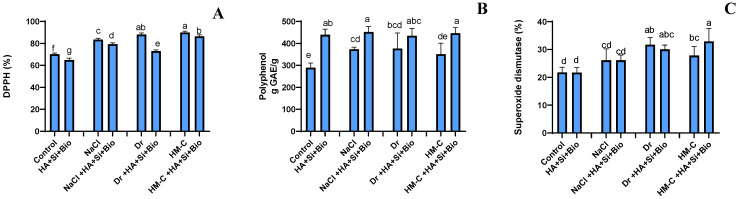
Bioassay of the antioxidant activity related to DPPH radical scavenging (**A**), polyphenol (**B**), and superoxide dismutase (**C**) production in the rice shoots. The error bars represent the standard deviation. Each data point represents the mean of at least three replicates. The bars with different letters are significantly different from each other at *p* ≤ 0.05. The abbreviations of the treatments are detailed in Table 2.

The superoxide dismutase (SOD) content was significantly increased by the SS (20%), DS (46%), and HM-C (28%) conditions compared with the control under NS conditions. The treatment with HA + Si + biochar showed no significant differences under the SS and DS conditions. However, the SOD levels were significantly elevated by 18% under the HM-C condition. The 2,2-diphenyl-1-picrylhydrazyl (DPPH) and polyphenol content were significantly increased under the SS, DS, and HM-C conditions. However, the treatment with HA + Si + biochar significantly reduced the DPPH levels and considerably increased the polyphenol content under all conditions (Figure 7).

#### 2.2.7. pH of the Soil and Treatment Solution

**Figure 8:**
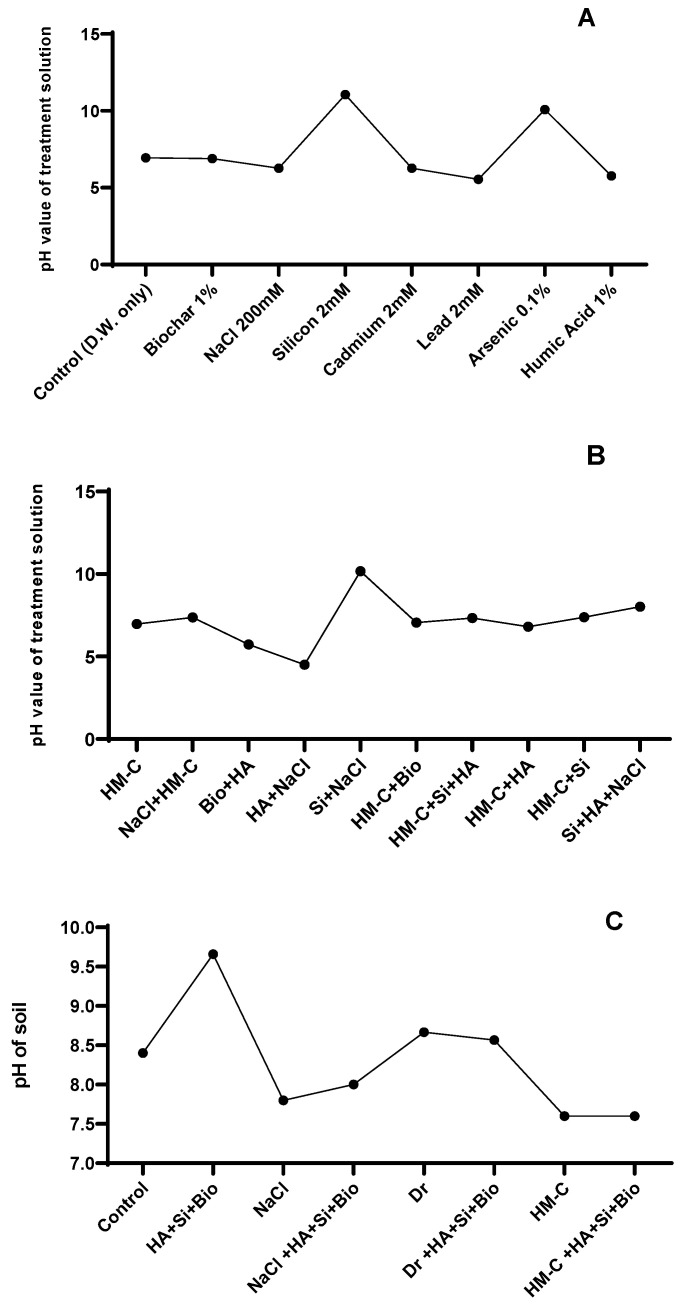
pH values of the treatment solutions spiked with HA, biochar, and silicon alone (**A**) and in combination (**B**) that can influence the soil properties. The pH value of soil after experiment (**C**). Each data point represents the mean of at least three replicates. The combination treatments in panel B were mixed according to the concentrations indicated in panel A. The abbreviations of the treatments are detailed in Table 2.

The HA + Si + biochar treatment increased the pH by 14% under normal conditions, whereas slight differences were observed in the other soil treatments. However, the pH of the treatment solution showed a significant pH elevation in the Si- and As-spiked solutions, whereas a lower pH level was observed in the HA-spiked solution (Figure 8). The treatment solution that was spiked with Si and As showed an increase in the pH level, whereas the solution spiked with HA showed a decrease in the pH level. Therefore, a specific dose selection is crucial to maintain soil health.

## 3. Discussion

The development of a comprehensive methodology and biofertilizer to manage multiple abiotic stresses, such as DS, SS, and heavy metal toxicity, in crops is crucial for achieving the goal of sustainable agriculture that is threatened by the increasing human population, climate change, and industrialization [36]. HA and biochar are the traditional biological tools used as biofertilizers. These tools are a rich source of Si, which has been identified as an essential nutrient for several crops, including rice [37,38]. In this study, we found that the HA + Si + biochar combination was effective in mitigating SS and DS and that the Si/biochar combination alleviated As, Pb, and Cd toxicity in rice (Appendix A). Here, we elucidate how these tools interfere with the metabolomics and molecular aspects of crops in terms of their endogenous phytohormones, osmotic balance through ion regulation, antioxidant activation, toxic radical scavenging, and metal-regulating transcriptomics.

The basic mechanism of action of the biological tool described in this study was that biochar and HA could replenish the soil organic matter and improve nutrient bioavailability for plant uptake, thus enhancing the water holding capacity of the soil, sustaining the microbiome, and conferring resistance to crops under SS and DS conditions [39,40]. In addition, biochar could detoxify heavy metals through metal stabilization and chelation and could serve as a potential source of Si, which is a vital element for rice and regulates various metabolic phenomena in crops [41]. Due to these fundamental functions, in our experiment, the HA + Si + biochar treatment significantly improved the plant morphological attributes by elevating the levels of essential nutrients, such as K, Ca, and Si, in rice plants.

Metal transporter genes play a key role in the translocation and deposition of mineral elements in a plant cell. The discovery of the Si influx and efflux transporters (Lsi1 and Lsi2) was a breakthrough in the understanding of rice genomics and metabolomics [42,43]. However, these genes also serve as major arsenic and other heavy metal transporters. Our results revealed the differential expression patterns of the metal transporter genes. They were highly expressed in DS conditions and remained neutral in SS conditions. However, their expression decreased under HM-C stress conditions when treated with the HA + Si + biochar combination. These results were further validated by the higher Si and As concentrations in the HA + Si + biochar-treated rice plants. Under heavy metal stress conditions, the lethality was more pronounced in rice crops because HA + Si + biochar triggered the hyperaccumulation of arsenic and cadmium. Arsenic is extremely toxic to crops, while Si is a major nutrient in silicon hyperaccumulating crops, such as rice, and follows the similar transport route that is mediated by the As and Si transporters (Lsi-1, Lsi-2, and Lsi-6) [44,45]. Additionally, the Cd concentration was also significantly increased following the HA + Si + biochar treatment, whereas the Pb concentration remained stable. Si mitigates several abiotic stresses, including heavy metals [46,47]. Therefore, it is apparent that the regulation of the Lsi family genes plays a vital role in the translocation of Si. On some occasions, the higher accumulation of Si combats toxicity by lowering the heavy metal uptake. Therefore, the exact interaction of Si, Lsi1, and Lsi2 under heavy metal stress remains unclear. Since the vitality and toxicity have similar mechanistic routes, a careful risk assessment in rice production appears inevitable.

The metal tolerance protein (MTP) genes are the specific transporters involved in the sequestration of heavy metal ions [48]. Similarly, the natural resistance-associated macrophage protein (NRAMP) gene is reported as the transporter of Fe and Cd in rice [49]. Our results showed that the expressions of OsMTP1 and OsNramp were significantly elevated in the stressed condition. In particular, OsMTP1 and OsNramp were reduced by the HA + Si + biochar treatments under HM-C stress. Our results were consistent with the findings of Das et al. [50], who demonstrated that OsMTP1 was upregulated in the seeds and leaves of rice shoots under Cd stress, which resulted in the reduction in phytotoxicity in crops. Similarly, Tiwari et al. [51] demonstrated that the OsNramp expression enhanced the tolerance of *Arabidopsis* to the increased accumulation of As and Cd. The expression of these metal transporter genes is highly affected by ion regulation to maintain cell homeostasis and the detoxification process [52].

The toxicity induced by the increased accumulation of Na^+^ and Cl^−^ and the efflux of K^+^ is an important phenomenon in salt-stressed plants [53]. A similar pattern of inducing toxicity was observed in our experiments, where the Na^+^ content was significantly elevated under SS in rice plants. However, the HA + Si + biochar combination treatment significantly increased the Ca^2+^ and K^+^ intake under all stress conditions and greatly lowered the Na^+^ influx in salt-stressed rice plants. The NHX family of genes contributes significantly by maintaining Na^+^ homeostasis in crops to confer stress tolerance [54]. The HA + Si + biochar-treated plants showed significant improvement in their plant growth attributes under salt and drought stress, where the expression of the Na^+^/H^+^ antiporter gene (*OsNHX1*) was considerably elevated. Our results were in agreement with several studies, such as that by Saidimoradi et al. [55], who reported that the treatment with HA, biochar, and Si improved the salt tolerance index by reducing the Na^+^ influx and enhancing K^+^ accumulation in strawberries, decreased the H_2_O_2_ and lipid peroxidation under DS in rice [56], and enhanced the microbial activity, antioxidants, soil organic matter, mineral nutrients (NPK), and yield of peanuts [29] and coriander [57].

The common free radical that triggers toxicity under all stressors is ROS [58]. In our results, we observed that melatonin was highly elevated under the HM-C stress conditions compared with the other stresses, which was reduced with the HA + Si + biochar treatment and resulted in a higher MDA content under HM-C stress. Melatonin enhanced the plant resistance directly by neutralizing ROS and indirectly by stimulating antioxidant enzyme activities, metabolites, and transcription factors [59]. A lower melatonin content under the HM-C condition may have resulted in higher lethality. These findings were supported by several studies, which reported that the application of exogenous melatonin mitigated the oxidative stress induced by heavy metals and other abiotic stresses [60,61]. The level of melatonin synthesis in crops is largely dependent on the concentration of metal ions, radicals such as H_2_O_2_ and NO, and environmental factors that regulate the enzymatic reaction pathways of tryptophan, tryptamine, and serotonin [62]. In addition, proline is directly connected to stomatal conductance through guard cell activity, which controls the transpiration rate and is a vital element in crops, especially under drought conditions, in combination with the endogenous phytohormone ABA, which plays a significant role in retaining higher water potential in crops [63,64]. The HA + Si + biochar treatment increased the proline content under HM-C and SS conditions, whereas it was reduced under DS conditions, which accounted for the reduced MDA content under salt and drought conditions.

The cross-talk of phytohormones, such as ABA and SA, is further interrelated with antioxidant activity [65]. In our experiments, the HA + Si + biochar treatment considerably reduced the ABA level and elevated the SA concentration. Moreover, antioxidant activities, such as polyphenol, were highly increased by the HA + Si + biochar treatment, whereas SOD and the DPPH radical scavenging activity demonstrated a differential pattern. In rice, a Ca^2+^/CaM-dependent protein kinase is essential to activate ABA and to subsequently trigger SOD and catalase (CAT) [66]. ABA and SOD have a potential cross-talk in defense through the conversion of O_2_ to H_2_O_2_, followed by other enzymes such as CAT and ascorbate peroxidase that stimulate the detoxification process of H_2_O_2_ to O_2_ to produce 2H_2_O [67]. In addition, the plant has a phenylpropanoid biosynthetic pathway for the synthesis of a wide array of phenolic compounds through the *PAL* (phenylalanine ammonia lyase) gene, which is also an initiator of SA to neutralize ROS through the chelation of metallic ions, the activation of endogenous phytohormones, and the improvement of absorption sites [68]. Additionally, the exudation of phenolic compounds from the roots sustains the microbiomes that enhance the plant–microbe interaction defense mechanism [69]. Polyphenols are also reported to trap DPPH, which is a stable free radical [70]. The DPPH radical scavenging activity in the rice shoots was highly activated. However, the mechanism that links the DPPH pros and cons against ROS and the other antioxidant enzymes, especially in plants under stress, lacked sufficient evidence.

The current findings revealed the pros and cons of HA and biochar when used in combination with Si in rice grown under SS, DS, and HM-C stress conditions. Moreover, it also addressed the approaches that were formulated by environmentalists and agriculturists in terms of heavy metal stress mitigation measures. The environmentalist’s fundamental approach is to replenish the soil by accelerating the metal uptake through crops, known as the phytoremediation process. Meanwhile, agriculturist approaches are based on the detoxification of crops by lowering the heavy metal accumulation in the crops. The findings of this current study could be used to develop appropriate guidelines for both approaches (Appendix A).

## 4. Materials and Methods

### 4.1. Experimental Work Plan

The Experiment 5 design was derived from the results obtained from Experiments 1–4. In this study, we included the physiological data from only Experiment 5 because it reflected the outcomes of the first four experiments (Table 3).

### 4.2. Plant Experiments

#### 4.2.1. Materials

Humic acid (FUJIFILM, Osaka, Japan), biochar (95% wood charcoal, 5% water; Gyeongdong Biochar, Yeongwol, Republic of Korea), arsenic (sodium meta arsenite; MW: 129.91 g·mol^−1^; SIGMA-ALDRICH, St. Louis, MO, USA), cadmium (cadmium sulfate hydrate; MW: 769.53 g·mol^−1^; Daejung Chemical and Metals Co., Ltd., Siheung-si, Republic of Korea), lead (lead II chloride; MW: 278 g·mol^−1^, SIGMA-ALDRICH), sodium chloride (NaCl; FW: 58.44; DUKSAN, Ansansi, Republic of Korea), silicon (sodium metasillicate pentahydrate; MW: 212.14 g·mol^−1^; SIGMA-ALDRICH), soil (coco peat, 25%; peat moss, 10%; vermiculite, 30%; sandy soil, 19%; diatomite, 10%; perlite, 3%; bottom ash, 2.5%; fertilizer, 0.48%; humectant, 0.02%; NongKyung, DOOBAENA PLUS, Jincheon, Republic of Korea).

#### 4.2.2. HA, Si, and Biochar Screening

The rice seedlings were prepared using paddy soil (NongKyung, DOOBAENA PLUS, Jincheon, Republic of Korea). Twenty-one-day-old seedlings of equal sizes were transplanted into a pot (400.87 cm^3^, radius: 6.2 cm, height: 12 cm, and shape: conical) containing 360 g of soil. After 2 days, they were treated with 1%, 2%, 3%, 4%, or 5% of HA and biochar and 1 mM, 2 mM, 3 mM, 4 mM, or 5 mM of Si. The biochar was ground to a fine powder before being applied to the soil using a mortar and pestle to enhance soil incorporation and bioavailability for plant nutrient uptake. Visual observation and the morphological attributes showed that 5 mM of Si had a burning effect on the rice shoots, which consequently reduced the plant’s growth characteristics. The biochar demonstrated optimum growth with no detrimental effects at any of the doses. Although HA showed no detrimental effects, it significantly lowered the pH level of the soil as the dose increased. Based on these observations, the optimum doses were selected as 2 mM Si, 1% HA, and 2% biochar for all the stress conditions.

#### 4.2.3. pH Level Measurement of the Treatment Solutions

The treatment solution was prepared according to the optimum doses that were selected as 2 mM of Si, 1% HA, and 2% biochar and used in this experiment. The pH was measured according to the experimental design, i.e., alone and in combination. In brief, 100 mL of double distilled (DD) water was placed in a flask, Si/HA/Bio were added in different combinations, and the pH levels were measured using a pH meter (Seven Compact^TM^ pH/Ion S220, Schwerzenbach, Switzerland).

#### 4.2.4. Plant Experiment with Different Stresses

The experiment was performed in the controlled greenhouse chamber (temperature; 28 ± 4 °C, relative humidity: 66% ± 6%) under natural daylight at Kyungpook National University, Daegu, Republic of Korea. The 3-week-old rice seedlings were prepared and selected as mentioned above during screening. The seedlings were primed for 8 h with HA and Si, whereas the biochar was incorporated before transplanting. Two equal-sized seedlings were transplanted into each pot containing 360 g of soil. Each treatment had at least 10 replicates. After 10 days of transplanting, 1% HA (50 mL; twice) and 2 mM of Si (50 mL; twice) were treated as the alone applications, whereas all the solutions were mixed according to the experimental design and used as the treatments for the combined application. After 1 week, the plants were subjected to 200 mM NaCl (50 mL day^−1^) and HM-C (0.1% As + 2 mM Cd + 2 mM Lead; 50 mL day^−1^) three times in Experiment 5. After 10 days, the plants were harvested and freeze-dried using liquid N_2_ for further biochemical analysis. Fresh samples were used to measure melatonin and for gene expression analysis. For the modeling of the drought stress condition, the method described by Adhikari et al. [71] with slight modifications was followed. The soil moisture level was maintained at 20–40% by calibrating a soil pH and humidity tester (Model DM-5, Takemura Electric Works, LTD., Tokyo, Japan). In brief, 50 mL of water per pot was supplied for the first 3 days, 40 mL per pot for the second 3 days, and 30 mL per pot for the last 4 days, to specifically induce drought stress. The chlorophyll content was determined using SPAD. The samples were harvested in the same manner as described for the SS and HM-C stress conditions.

### 4.3. Biochemical Analysis of Rice Plants

#### 4.3.1. Endogenous Phytohormone Analysis

**ABA**: To extract and quantify ABA, the method described by Kim et al. [72] was used. In brief, isopropanol and acetic acid (95:5 *v*/*v*) were used to extract 0.5 g of lyophilized powdered material. Gas chromatography with mass spectrometry and selected ion monitoring (GC-MS/SIM) (6890N Network GC System and 5973 Network Mass Selective Detector; Agilent Technologies, Santa Clara, CA, USA) was used to evaluate the ABA extracts. The obtained peak was compared with the standard curve that was developed from the internal ABA standard (0, 3, 5, 5, 7, 7, 7-d6).

**SA**: The method described by Seskar et al. [73] was used to extract and quantify the endogenous SA. In brief, a freeze-dried sample was extracted with 90% MeOH, centrifuged (9660× *g*; 4 °C), and the supernatant was collected and concentrated using a speed vac (SPD2030; Thermo Fisher Scientific, Waltham, MA, USA), followed by the addition of 5% trichloroacetic acid. The solution was centrifuged, and the supernatant was treated with an extraction solution containing a 49.5% cyclopentane:ethyl acetate:isopropanol (ratio 49.5:49.5:1 (*v*/*v*)), dried with N_2_ gas, and recovered with MeOH. The peaks were obtained using high-performance liquid chromatography (HPLC) at a flow rate of 1.0 mL/min in a C18 reverse-phase HPLC column (HP hypersil ODS, particle size 5 m, pore size 120 Waters; size 3.9 × 300 mm).

#### 4.3.2. Mineral Elemental Analysis

The method described by Adhikari et al. [71] was used to quantify the mineral elements Si, P, K, and Na. In summary, 0.1 g of freeze-dried material was suspended in a concentrated HNO_3_ reagent and heated on a hot plate using the Ultrawave (milestone) microwave digestion equipment. The sample was sonicated, separated, filtered, and diluted using ddH_2_O, and the resulting solution was used to inject and measure the sample using an ICP-MS analyzer (Optima 7300DV, Perkin-Elmer, Akron, OH, USA).

#### 4.3.3. Proline Quantification

The freeze-dried plant sample was hydrolyzed in 6 N HCl for 24 h under a vacuum, first at 110 °C, then evaporated at 80 °C, and recovered with 0.02 N HCl followed by filtration (0.45 μm Millipore filter DISMIC-25CS, ADVANTE, Tokyo, Japan). The quantification of proline was performed using an automatic amino acid analyzer (HITACHI Corporation L-8900, Tokyo, Japan) connected to a HITACHI HPLC system (packed column with ion-exchanging resin, No. 2622 PF; 4.6 60 mm) and an ultraviolet detector (VIS1: 570 nm, VIS2: 44 nm).

#### 4.3.4. Extent of the Lipid Peroxidation MDA Analysis

The MDA content in the rice shoots was measured using the procedure described by Gupta et al. [74]. In brief, 10 mL of 5% trichloroacetic acid was used to extract 0.5 g of a fresh leaf sample, and the supernatant was separated. The solution was suspended in thiobarbituric acid and then placed in a water bath at 85 °C for 25 min. The sample was quickly chilled to 4 °C using ice. After filtering the extract, the absorbance was measured at 600 nm and 532 nm using a spectrophotometer (EPOCH 2 microplate reader, Winooski, BioTek Instruments, Inc., Winooski, VT, USA).

#### 4.3.5. cDNA Synthesis and Real-Time PCR for the Analysis of the Metal Transporter and Melatonin Pathways

The total RNA was isolated using a commercial RNA extraction kit (BIOFACT, Daejeon, Republic of Korea), according to the manufacturer’s instructions. The BioFACT RT-Kit (BIOFACT) was used to synthesize the first-strand cDNA from 1 g of RNA. A three-step qRT-PCR was performed using the CFX Duet Real-Time PCR system (BIO-RAD, Hercules, CA, USA). The primers that were used are provided in the Supplementary Table S1.

#### 4.3.6. Melatonin Quantification

The method described by Kwon et al. [75] was followed to detect and quantify the melatonin content in the rice shoots using a commercial Melatonin ELISA Kit (Enzo Life Sciences, Inc., Farmingdale, NY, USA). A lyophilized ground-up sample was homogenized with 1 × stabilizer and cold ethyl acetate (125 µL and 750 µL, respectively), centrifuged, and the obtained layer was separated and air-dried using N_2_. The pellets were suspended with the stabilizer, followed by a melatonin tracer and antibody treatment with a melatonin-conjugated solution and 3,3′,5,5′-tetramethylbenzidine (TMB) substrate. The optical density of the contents was measured at 450 nm using a Multiskan GO UV/V microplate spectrophotometer (Thermo Fisher Scientific, Waltham, MA, USA).

#### 4.3.7. Antioxidant Activity Assay (DPPH and Polyphenol)

To determine the SOD, DPPH, and polyphenol contents, a spectrophotometer (EPOCH 2 microplate reader, BioTek Instruments) was used to measure the absorbance at 517 nm, 420 nm, and 750 nm, respectively. The samples were extracted using 100% methanol, and the protocols described by Wang et al. [76], Marklund [77], and Adhikari et al. [78] were followed to measure the DPPH, SOD, and polyphenol, respectively.

### 4.4. Statistical Analysis

The data were analyzed using R Studio (version 4.3.1). A least significant difference test (*p* < 0.05) was used to determine the significant differences among the treatments. The mean and standard deviation were calculated using Microsoft Excel 2019, and the data were graphically presented using GraphPad Prism (version 8; San Diego, CA, USA).

## 5. Conclusions

Overall, the HA + Si + biochar treatment had a significant positive effect, especially in mitigating drought and salinity stress. The HA used alone or in combination treatment increased the heavy metal uptake and ROS level. Although HA imparted a negative result, these findings also enhanced the scope of HA application by improving the phytoremediation process by using the heavy metal resistance of plants. Overall, the current study provides a comprehensive baseline for selecting an agricultural tool to mitigate particular stresses that are present in crops and to achieve the goal of sustainable agriculture.

## Figures and Tables

**Table 2:** Experiment 5 (combined treatment and stress conditions).

No-Stress Condition (NS)	
T1	Control
T2	HA + Si + biochar
**Salt-Stressed Condition (SS)**	
T3	NaCl only
T4	NaCl (HA + Si + biochar)
**Drought-Stressed Condition (DS)**	
T5	Dr only
T6	Dr (HA + Si + biochar)
**Combined Heavy Metal Stress Condition (HM-C)**	
T7	HM-C only
T8	HM-C (HA + Si + biochar)

**Conditions: NS**, No-Stress; **SS**, Salt stress; **DS**, Drought Stress; **HM-C**, Combined heavy metal (As + Pb + Cd) stress; **Dr**, Drought; **NaCl**, Salt. **Treatment tools: HA**, Humic acid.

**Table 3:** Experimental work plan.

Experiment 1 (NaCl)	Experiment 2 (Arsenic)	Experiment 3 (Drought)	Experiment 4 (As, Pb, and Cd)
		**No-Stress Condition (NS)**	
Control	Control	Control	Control
HA	HA	HA	HA
Si	Biochar	Si	Si
HA + Si	HA + Biochar	HA + Si	HA + Si
		**Stressed Condition (SS, DS, and HM-C)**	
NaCl	As	Drought	HM-C
HA + NaCl	HA + As	HA + Drought	HA + HM-C
Si + NaCl	Biochar + AS	Si + Drought	Si + HM-C
HA + Si + NaCl	HA + Biochar + As	HA + Si + Drought	HA + Si + HM-C

**Conditions**: **NS**, no stress; **SS**, salt stress; **DS**, drought stress; **HM-C**, combined heavy metal (As + Pb + Cd) stress. **Treatment tools: HA**, humic acid.

## Data Availability

The data will be made available upon request.

## Supplementary Materials

The following supporting information can be downloaded at: https://www.mdpi.com/article/10.3390/ijms242417369/s1.

## Appendix A

From Exp 2 and 4 (heavy metal stress), we observed that the sole application of HA could significantly induce rice toxicity and ultimately led to death, which was mainly due to accelerating the HM uptake resulting in high oxidative stress. Meanwhile, the application of Si and Bio in combination or solely could prolong the survival duration of crops. From this observation, it was apparent that the presence of HA could trigger toxicity under HM stress. At the initial stage, the Si + Bio + HA-treated crops showed considerable resistance whereas, under an extended period, the Si + Bio + HA-treated application showed a higher mortality than that of the HM-C-only-treated plants. From these findings, HA could be employed for the phytoremediation process whereas Si and Bio could be applied for detoxification and boosting immunity in rice under heavy metal stress. Similarly, Si, Bio, and HA could be applied in assemblance to mitigate drought and salt stress in crops.
