# Supplementary material for: Dynamics of Humic Acid, Silicon, and Biochar under Heavy Metal, Drought, and Salinity with Special Reference to Phytohormones, Antioxidants, and Melatonin Synthesis in Rice
Source: Int J Mol Sci. 2023 Dec 11;24(24):17369. doi: 10.3390/ijms242417369 (PMC10743973; doi:10.3390/ijms242417369)
Supplement: Supplementary file 1 [file ijms-24-17369-s001.zip › ijms-2733773-supplementary.pdf]

Supplementary Table 1. Primers used in the gene expression analysis

Primer	Forward	Reverse
**OsLSi1**	ACGAGATGTCGTCGATCGTG	GCACACGCCCAAAATTAGCA
**OsLSi2**	TATGTTCTCCGACATCTCCAGC	GCAAGATCACCTTCCCCAAGT
**osNramp1**	CAGAAAGGAGCCAGCATGGA	AGAGCCAGCAATGAGAACCC
**OsMTP1**	ATGGACAGCCATAACTCAGCACCTCCCC	CTA CTC GCG CTC AAT CTG AAT GGT TC
OsNHX1	AGCGGCATTCTCACCGTATT	GAGCAATCGACACAGCTCCT
**OsACT1**	TGAATCTGGTCCAGGCATCG	TGGGACGCATGCAAACAATC
